# i-cisTarget 2015 update: generalized cis-regulatory enrichment analysis in human, mouse and fly

**DOI:** 10.1093/nar/gkv395

**Published:** 2015-04-29

**Authors:** Hana Imrichová, Gert Hulselmans, Zeynep Kalender Atak, Delphine Potier, Stein Aerts

**Affiliations:** Laboratory of Computational Biology, Center for Human Genetics, University of Leuven, 3000 Leuven, Belgium

## Abstract

i-cisTarget is a web tool to predict regulators of a set of genomic regions, such as ChIP-seq peaks or co-regulated/similar enhancers. i-cisTarget can also be used to identify upstream regulators and their target enhancers starting from a set of co-expressed genes. Whereas the original version of i-cisTarget was focused on *Drosophila* data, the 2015 update also provides support for human and mouse data. i-cisTarget detects transcription factor motifs (position weight matrices) and experimental data tracks (e.g. from ENCODE, Roadmap Epigenomics) that are enriched in the input set of regions. As experimental data tracks we include transcription factor ChIP-seq data, histone modification ChIP-seq data and open chromatin data. The underlying processing method is based on a *ranking-and-recovery* procedure, allowing accurate determination of enrichment across heterogeneous datasets, while also discriminating direct from indirect target regions through a ‘leading edge’ analysis. We illustrate i-cisTarget on various Ewing sarcoma datasets to identify EWS-FLI1 targets starting from ChIP-seq, differential ATAC-seq, differential H3K27ac and differential gene expression data. Use of i-cisTarget is free and open to all, and there is no login requirement. Address: http://gbiomed.kuleuven.be/apps/lcb/i-cisTarget.

## INTRODUCTION

The field of regulatory genomics is generating vast amounts of sequencing data related to transcription factor binding, chromatin activity and gene expression. Whereas many tools are available for the functional analysis of gene signatures, such as Gene Ontology enrichment analysis (1–3) and for the identification of enriched transcription factor motifs in co-expressed gene sets (4–8), fewer web tools exist to analyse sets of genomic regions. Different types of post-processing and functional analysis of a set of genomic regions can be used to gain insights into regulatory and functional relationships. Firstly, motif discovery identifies transcription factor binding sites and predicts new regulators and co-factors. Tools exist for *de novo* motif discovery (e.g. PeakMotifs (9), MEME (10)) and for the enrichment analysis using libraries of position weight matrices (e.g. oPOSSUM-3 (4), the SeqPos tool in Cistrome (11) and Homer (7), although the latter is only available command-line). A second question one can ask for an experimentally derived set of genomic regions is whether it correlates with existing ChIP-seq or chromatin activity data such as histone modifications or open chromatin (DNaseI hypersensitivity, FAIRE-seq, ATAC-seq). An example tool that performs such correlations is the ENCODE ChIP-Seq Significance Tool (12). A third kind of analysis that is often performed on genomic regions is to associate each region to one or more candidate target genes and analyse the function (e.g. by GO (13)) of the resulting target gene set. Such a procedure is implemented by the web tool GREAT (14).

i-cisTarget aims at combining motif and track enrichment in a single analysis through a unified statistical framework and goes beyond existing tools concerning the amount of candidate position weight matrices and the number of experimental data tracks tested. In this article we present a major update of i-cisTarget, now including support for human and mouse datasets; increasing our motif collection to nearly 10,000 PWMs; and adding human and mouse specific databases with more than 4000 regulatory data tracks. One of the challenges is to make these analyses computationally tractable, so that they can be run in a web tool. To this end, we generated collections of candidate regulatory regions (CRRs) for the human and mouse genome. These regions are scored and *ranked* offline, so that the online *recovery* analysis becomes highly efficient. The output of i-cisTarget are predictions of key transcription factors alongside a prioritized list of direct transcriptional targets and the actual cis-regulatory modules (CRM) and transcription factor binding sites.

## MATERIALS AND METHODS

### Regulatory regions and data sources

#### Defining candidate regulatory regions for the human, mouse and fly genome

We defined sets of CRRs for the human, mouse and fly genomes. To delineate human CRRs the following publicly available regulatory data were used (see Table 1A): DNAseI Hypersensitive (DHS) uniform clustered peaks across 125 cell lines from ENCODE (15), General Binding Preference models (16), regulatory elements from ORegAnno (17), VistaEnhancers (18), predicted cis-regulatory modules (19), CpG islands and proximal promoters (both downloaded from UCSC table browser (20)), conserved non-coding sequences (CNS) and ultraconserved elements (UCR). For mouse CRRs the same features (mouse genome) were used except General Binding Preference models, using ultra-conserved non-coding elements (21). DHS peaks in mouse cell lines were used (22) (Table 1B). Where needed the UCSC *liftover* tool (23) was used to convert genome coordinates to hg19 and mm9.

**Table 1.** Publicly available regulatory datasets used to create i-cisTarget human CRRs (A) and publicly available regulatory datasets used to create i-cisTarget mouse CRRs (B)

**A**.									
	GBP	CpG	Proximal promoters	CNS	UCR	Oreganno	Vista enhancers	CRMs	DHS
Number of regions	61550	27718	34722	232101	15931	23112	1339	123500	1281988
% of the genome	1.77	0.73	0.67	2.25	0.13	0.39	0.07	2.05	13.36

**B**.									
	CpG	Proximal promoters	CNS	UCR	Oreganno	Vista enhancers	CRMs	UCNE	DHS
Number of regions	16026	22984	231478	15927	16976	339	91176	4335	14971709
% of the genome	0.40	0.41	2.49	0.14	0.38	0.02	1.66	0.05	29.16

Next, all these features were merged and regions having an overlap of at least 20% with insulator elements or at least 80% of coding exons were removed. Next, regions with an overlap <20% with insulators or 80% with exons are split and the regions containing the insulator or coding exons were removed. Remaining regions are then filtered based on size and regions shorter than 30 bp are removed. Finally, any resulting regions shorter than 1000 bp were extended if possible to 1000 bp in a direction that prevents overlap with an insulator or exon. The complete procedure of creating CRRs yielded 1,223,024 and 938,376 regions for human and mouse, respectively (representing ∼35% of the human genome and ∼42% of mouse genome). The sizes of the regions are relatively short, with more than 96% of human and 93% of mouse regions being smaller or equal than 1000 bp. The procedure of creating the fly CRRs (136,353 regions) is described in (24). The sets of CRRs per species are provided as bed files on the i-cisTarget website.

### Mapping user input to candidate regions

**Figure 1.**
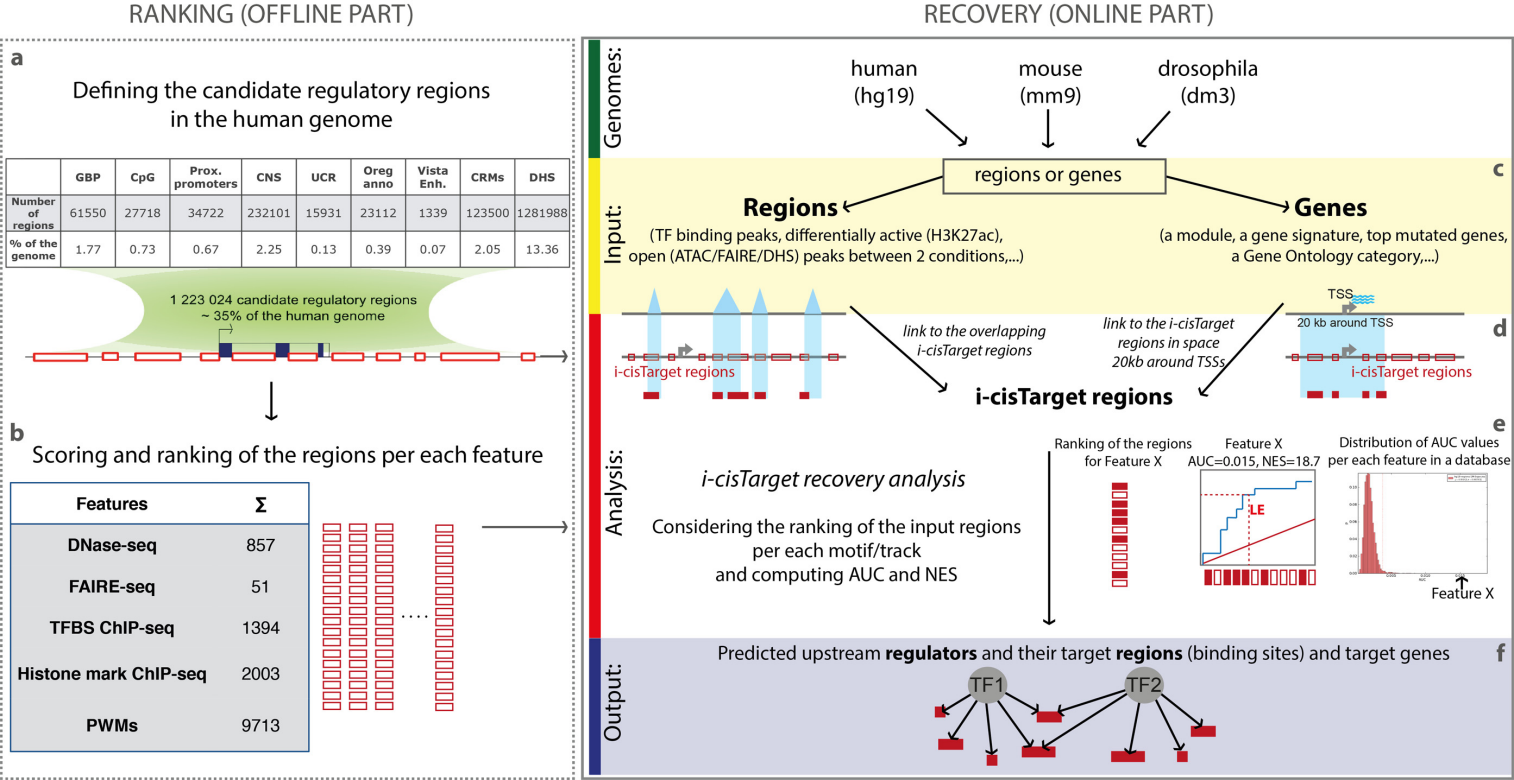
i-cisTarget workflow. The i-cisTarget web-tool consists of two parts, namely the *ranking* (offline part) and *recovery* (online part). (**a**) A set of 1,223,024 candidate regulatory regions (CRRs) is defined based on publicly available regulatory data, representing 35% of the human genome. (**b**) The collection of CRRs is scored and ranked according to different features, including motifs, TF and histone ChIP-seq, DNase-seq and FAIRE-seq, resulting in large ranking databases. (**c**) The online part starts with user input, which can be a set of genomic regions or a set of genes for human, mouse or fly. (**d**) The input set is mapped to the candidate i-cisTarget regions. In the case of regions/peaks, the overlapping CRRs (minimum overlap percentage is a parameter) with the peaks are considered in the analysis. When a gene set is used as input, then CRRs overlapping the entire space of X kb around TSSs are taken into the analysis (the default space for human and mouse genome is 20 kb around TSS; the default for fly is 5 kb upstream of TSS and all introns). (**e**) The recovery analysis identifies the feature for which the input CRRs are most enriched in the top of the CRR ranking of that feature. This enrichment is calculated by the Area Under the recover Curve (AUC) and all features with normalized AUC (i.e. Normalized Enrichment Score, or NES) above 3.0 are returned. (**f**) For each enriched feature and upstream regulator the direct target regions are provided, with a link to a BED file for download and a track in the UCSC Genome Browser.

The input can be either a set of co-regulated genomic loci, such as ChIP-seq or DHS/FAIRE/ATAC-seq peaks; or a set of co-expressed genes. In both cases the input is linked to the CRRs (as shown in the Figure 1c and d). Peaks/loci are converted to the overlapping CRRs according to the ‘fraction of overlap’ parameter (specified by the user) representing the overlap between the input peaks and i-cisTarget CRRs. For example, if the fraction of overlap is set to 0.4 (default) then only i-cisTarget regions that overlap at least 40% with the input peaks will be used in the analysis. On the Report page we provide a text file with the overlap details of the input regions with i-cisTarget CRRs. If the input is a set of genes, then the input genes are linked to CRRs by collecting all CRRs located in the neighbourhood of a gene. This neighbourhood extends upstream and downstream of the transcription start site (TSS) and is a parameter that can be selected by the user. For human and mouse the default space is 20 kb around TSS and for fly it is set to 5 kb upstream of TSS and all introns.

### Region-scoring and ranking

#### Scoring of candidate regulatory regions with position weight matrices

The motif collection was compiled from several resources which are described in (8) and yielded a large collection of 9713 position weight matrices (PWMs or motifs). The predefined CRRs were scored using Cluster-Buster (25) for each PWM across 10 mammalian genomes, namely: hg19 (Homo Sapiens), bosTau4 (Bos Taurus), canFam2 (Canis familiaris), mm9 (Mus musculus), monDom5 (Monodelphis domestica), panTro2 (Pan troglodytes), ponAbe2 (Pongo pygmaeus abelii), rheMac2 (Macaca mulatta), rn4 (Rattus norvegicus) and susScr2 (Sus scrofa). The orthologous regions between mammalian genomes were obtained using *liftOver* tool (23). This process is similar to the one used for motif scoring of fly CRRs as described in (24) and for human/mouse genes in the iRegulon Cytoscape plugin (8). Regions were ranked for each genome separately and subsequently the rankings were aggregated into one final ranking per PWM using *OrderStatistics* (26,27).

### Scoring of candidate regulatory regions with experimental data tracks

Collections of human and mouse CRRs were also scored by the regulatory features: DHS, FAIRE, TF and histone ChIP-seq from ENCODE (15), Roadmap Epigenomics Project (28), data generated by Taipale laboratory (29), Aerts laboratory (H3K27ac, H3K27me3, FAIRE and MITF ChIP-seq on short-term melanoma cultures generated in-house (30) as well as p53 ChIP-seq and FAIRE on MCF-7 (8)) and Mouse ENCODE (22). Used feature types and resources are mentioned in Tables 2 A and B. Concerning the tracks from ENCODE, all the available replicates were used. For the scoring the maximum score of broad or narrow peaks (signalValue column in bed file format) within a region was used. Finally, each region has one score per track. Taipale datasets were converted from hg18 to hg19 using *liftOver* tool (23) and fold enrichment value was used for the ranking. Bigwig files were used for the ranking of the CRRs for tracks from Roadmap Epigenomics Project. Finally, three separate databases of rankings were created: DHS and FAIRE, histone modifications and TF ChIP-seq.

**Table 2.** Human regulatory tracks included in the databases (A) and mouse regulatory tracks included in the databases (B)

**A**.					
	ENCODE	Epigenome roadmap	Taipale	Aerts	∑
DHS	467	390	0	0	857
FAIRE	37	0	0	14	51
Histone	402	1572	3	26	2003
TF ChIP-seq	1274	0	117	3	1394
∑	2180	1962	120	43	4305

**B**.					
	ENCODE				
DHS	150				
FAIRE	0				
Histone	209				
TF ChIP-seq	206				
∑	565				

### Recovery analysis

The i-cisTarget enrichment analysis is based on calculating the cumulative recovery of the input regions along each ranking in the selected i-cisTarget databases, similarly to gene set enrichment analysis (GSEA)-like approaches (31). Briefly, the rank positions of the user input (mapped to i-cisTarget CRRs) is considered for each motif/track ranking and the Area Under the recovery Curve (AUC) of these foreground regions is calculated. We are mainly interested in regions that are highly ranked and therefore we calculate the AUC for only a fraction of the top ranked regions. This fraction is defined by AUC threshold parameter, which is set by default at 0.5% (6115 and 4692 regions) for human and mouse, at 1% for fly genome (1364 regions). The raw AUCs are then normalized to a Normalized Enrichment Score: NES = (AUC-μ)/σ, where μ represents the mean of all AUC scores across all features in the corresponding database and σ represents standard deviation of all these AUC scores. Finally, the optimal threshold (or ‘leading edge’) in the recovery curve represents the optimal set of target regions and corresponds to the rank position (x-axis) at which the difference of recovered regions (y-axis) with the average number of recovered regions plus two standard deviations is largest; this is illustrated in Figure 1e. In this way the input set is divided into true positive sets (sets of cis-regulatory elements where upstream regulators bind; the input regions within the LE subset) and false positives (the input regions outside the LE subset). Note that since the analysis based on the entire PWM database can take several minutes (also depending on the input dataset), we implemented an option to run a quick analysis, based on the JASPAR motif collection (1312 PWMs) (32) and a more stringent NES threshold (NES > 4.0). These quick jobs are prioritized in the job queue so that preliminary results can usually be obtained within ∼20 s, whereas a full job may take around 5 min.

### Motif2TF mapping and motif clustering

Motif2TF mapping is used to associate the enriched motifs with candidate transcription factors. This mapping is based on a motif-TF network including direct evidence, orthology and motif–motif similarity as described in (8). Moreover, similar enriched motifs are clustered together by STAMP (33) which measures similarity using the sum of squared distances (SSD) and finds the optimal number of clusters using Calinski & Harabasz statistics. Then the motifs from the same cluster are marked in the same colour in the online report.

### Distinctive features compared with other tools

i-cisTarget is different from other tools in several regards. Only few existing tools provide an enrichment analysis of regulatory data tracks. The few tools that analyse track data, such as the ENCODE ChIP-Seq Significance Tool (12) are limited to TF ChIP-seq tracks only and do not include histone modifications or chromatin accessibility. In addition, to our knowledge no other tool combines track discovery with motif discovery in one single analysis, allowing to compare tracks and motifs, and to combine them into a single set of robust target predictions. Another key difference with other existing tools is the very large motif collection that is used for enrichment analysis, with nearly 10,000 PWMs, collected from various sources and species, clustered and mapped to human/mouse/fly transcription factors either by direct annotation, orthology and/or similarity. i-cisTarget also uses a highly robust statistical analysis––across multiple species––to calculate the feature enrichment. Contrary to most other tools this enrichment is not computed by a hyper-geometric statistical test, but rather by a *ranking-and-recovery* analysis. Moreover, i-cisTarget offers a unique feature that is the possibility to analyse both genes as well as regions or peaks as input allowing the user to analyse various datasets (e.g. differentially expressed or co-expressed genes; ChIP peaks; differentially active regions; CRM driving similar expression patterns). Also the output of i-cisTarget provides both a list of predicted target regions/enhancers and a list with the closest genes to these target regions. Another interesting feature that we added in the new version is a follow-up tool to compare two analysis results, even between different species, to identify commonly (or distinctively) found motifs that are enriched in both sets. Finally, many small details make i-cisTarget user friendly, such as the selection of the optimal subset of direct target regions from the input; the possibility to combine several motifs and tracks into a single new ranking; the availability to visualize predicted target regions as a custom track in the UCSC genome browser together with the motif and CRM predictions; and the possibility to export a Simple Interaction File (SIF) with motifs/tracks, target regions and the closest genes, that can be imported into Cytoscape (34) to be visualized as a network.

## WEB SERVER

We illustrate various types of analyses that can be performed by the i-cisTarget web tool using a case study on Ewing sarcoma (35). Ewing sarcoma is a cancer of bone and soft tissue and is characterized by chromosomal translocations involving *EWS* gene and transcription factors of the ETS family (ERG, FEV, FLI1, ETV1 and E1AF). EWS-FLI1 fusion product is the most common type as it is found in 90% of the cases (36). Riggi *et al*. performed ChIP-seq against the chimeric transcription factor EWS-FLI1, as well as ATAC-seq, RNA-seq and ChIP-seq against H3K27ac, all before and after EWS-FLI1 perturbations. Each type of profiling will be used in turn as input to i-cisTarget with the aim of identifying EWS-FLI1 direct target genes, regulatory regions and binding sites.

### Analysis of ChIP-seq peaks

i-cisTarget can be used for motif and track discovery on a set of ChIP-seq peaks of a particular transcription factor. Such analysis is usually performed as a quality control step of ChIP-seq data analysis, because the motif of the ChIP'ped transcription factor is expected to be enriched among the binding peaks. In addition, i-cisTarget uses the cross-species motif scores to optimally discriminate between directly bound regions and indirectly bound regions (or false positives). Finally, the motif and track analysis of ChIP-seq peaks can be useful to identify candidate co-factors of the ChIP'ped factor, in case the ChIP'ped regions are enriched for motifs or ChIP-seq peaks of other transcription factors.

**Figure 2.**
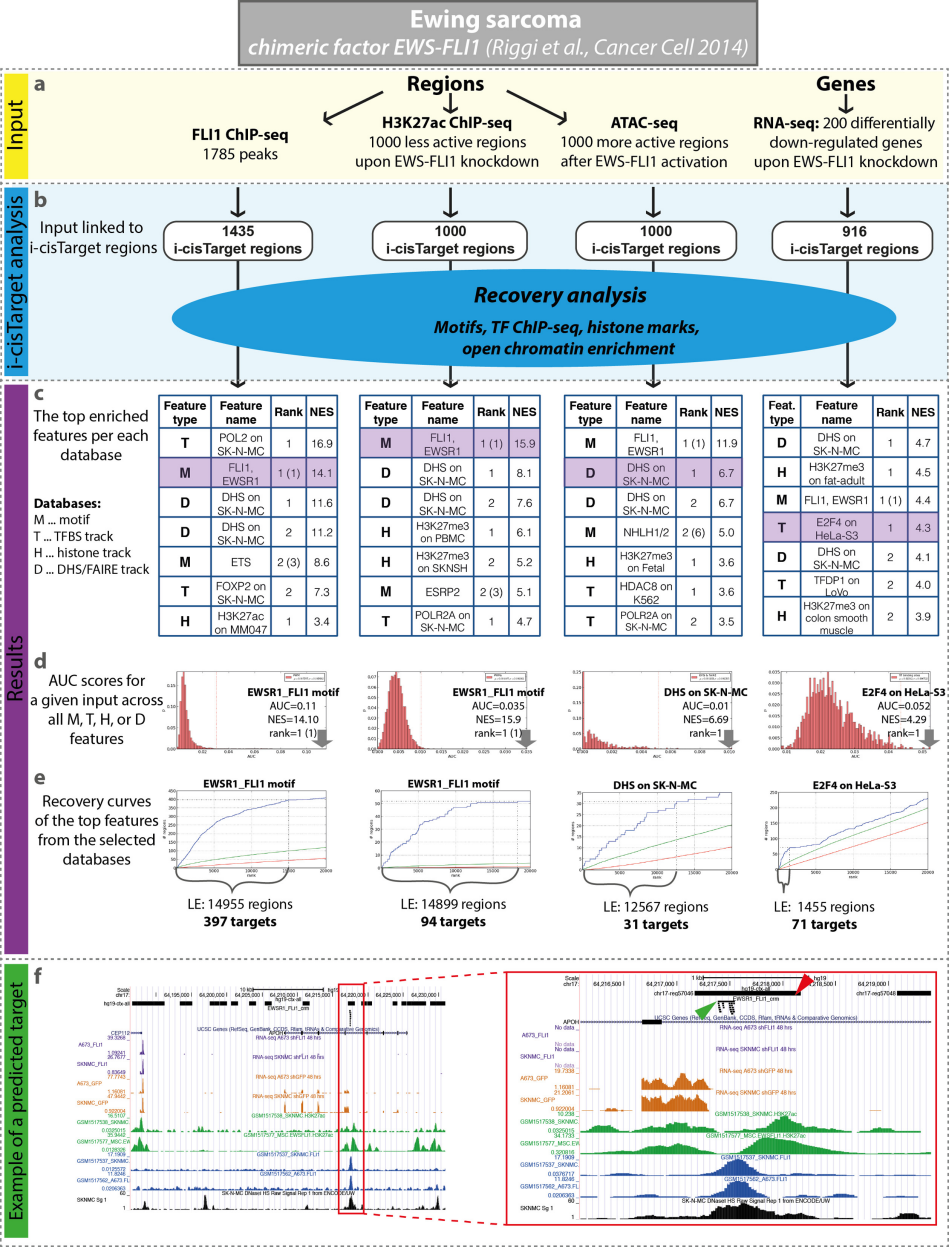
Ewing sarcoma case study. Various i-cisTarget analyses with different types of input, all related to EWS-FLI1 targets in Ewing sarcoma. (**a**) All input datasets are derived from Riggi *et al*. (35) and include FLI1 ChIP-seq peaks, top differentially less active peaks based on H3K27ac ChIP-seq upon EWS-FLI1 knockdown, differentially more open regions based on ATAC-seq after EWS-FLI1 activation and differentially downregulated genes after EWS-FLI1 knockdown (see ‘Materials and Methods’ section). (**b**) Input regions are automatically mapped to CRRs. (**c**). Each of the sets was analysed independently and reassuringly the expected motif EWS-FLI1 was ranked at the top, alongside regulatory tracks, mainly obtained on the SK-N-MC Ewing sarcoma cell line. Note that the rank of the motifs is represented by two values––the first one for the rank of the cluster of similar motifs, the second one is between brackets and represents the rank of the specific motif. (**d**) Distributions of AUC scores for a given input across all features in the selected databases (marked in purple in tables (c)) with an arrow indicating the top feature within that database. (**e**) The recovery curves for the top ranked features within the database, where the leading edge (LE) indicates the number of highly ranked target regions. (**f**) UCSC genome browser screenshot representing an example of one direct target region (red arrowhead) in the intron of gene APOH, which is included in the set of downregulated genes. This region was predicted as a target of EWS-FLI1 in i-cisTarget analyses, of the top less active H3K37ac peaks and the top 200 downregulated genes as well as FLI1 ChIP-seq peaks. The specific binding site is represented by a cluster of EWS-FLI1 motifs (green arrowhead), which was generated by i-cisTarget subsequent analysis, when the predicted target regions of EWS-FLI1 were scanned for CRMs of this factor. All these tracks are represented on the screenshot (from top to bottom): the CRRs, the predicted cluster of EWS-FLI1 motifs, RNA-seq peaks in SK-N-MC and A673 after shFLI1 (two purple tracks, published in (35)) and control (two orange tracks, published in (35)), H3K27ac peaks in SK-N-MC and MSC cell lines expressing EWS-FLI1 (green tracks, published in (35)), FLI1 ChIP-seq track in SK-N-MC and A673 cell lines (blue tracks, published in (35)) as well as DHS on SK-N-MC which was found as the top track within non-TF regulatory tracks (black track, from ENCODE database (15)).

As an example we use the ChIP-seq peaks of EWS-FLI1 as input. The authors ChIP'ped the C-terminal portion of FLI1 gene in two Ewing sarcoma cell lines (A673 and SK-N-MC) and defined the 1785 peaks that are present in both cell lines as the ‘core set of EWS-FLI1’ binding sites (provided in the Supplementary Table S1 of the corresponding paper). i-cisTarget analysis on this core set of binding sites identified the motif of the fusion product as the first motif (Figure 2c, i-cisTarget results on the website). Interestingly, ENCODE tracks of PolII ChIP-seq and DNAseI-seq on the Ewing sarcoma cell line SK-N-MC are highly ranked in the results. In addition, the remaining enriched features indicate a vast presence for ETS-family transcription factor motifs and this is indeed in agreement with the authors’ claim that the EWS-FLI1 oncogenic protein displaces ETS-family members from their native binding sites.

We also applied i-cisTarget to a previously published benchmark dataset of 36 ChIP-seq datasets selected from FactorBook (37). i-cisTarget identified the correct motif of the ChIP'ped TF for 27 (the motif ranked among the top five motifs, from which for 22 sets are the motifs ranked as the first) of the 36 datasets, when using the top 500 peaks as input. Interestingly, i-cisTarget can also be applied to the entire set of ChIP-peaks (sometimes several thousands of peaks) with the same computational efficiency. As expected, such input is slightly more noisy and yields the correct motif ranked at the first position for 18 of the 36 factors (19 correct motifs ranked among the top five motifs). The results from this analysis are available on the website.

In conclusion, when TF ChIP-seq peaks are analysed, i-cisTarget identifies the motif of the ChIP'ped TF, allowing for quality control, but also for the identification of co-factors and for distinguishing between direct and indirect targets.

### Analysis of other regulatory regions

The main advantage of i-cisTarget is the detection of upstream regulators for the regions that are found to be active (or alternatively, repressed), when the binding regulator is not known. To demonstrate this, we analysed the differentially H3K27-acetylated regions upon EWS-FLI1 knockdown in SK-N-MC and A673 cell lines, measured by H3K27ac ChIP-seq. For this, coverage of i-cisTarget regions was quantified using BEDTools (38) and differentially active regions after FLI1 knockdown (hence sites activated by EWS-FLI1) were identified using DESeq2 (39). The top 1000 (ranked on signed-log10(adjusted *P*-value)) regions that become less active upon EWS-FLI1 knockdown contained the EWS-FLI1 motif as the top ranked motif among all the 9713 motifs, indicating that many of these are directly targeted by EWS-FLI1 (see Figure 2c or results on the i-cisTarget website). We applied the same analysis on ATAC-seq data representing differentially more open regions after EWS-FLI1 activation in mesenchymal stem cells. Again, the top 1000 i-cisTarget regions were found to be enriched with the same EWS-FLI1 motif (see Figure 2c). On the other hand, regions that are activated upon EWS-FLI1 knockdown contained strong enrichment for ETS family factors, especially for ELF1 and GABPA. Such competition was also proposed by Riggi *et al*. (results available on the i-cisTarget website).

We furthermore applied i-cisTarget to various other sets of regulatory regions, such as similar enhancers that are active in the same cell type, DHS regions of a particular tissue or cell type, p300 bound regions in a tissue and others. The results of these analyses are available as examples on the i-cisTarget website.

### Analysis of gene signatures

To demonstrate the use of i-cisTarget on human co-expressed gene sets we re-analysed the RNA-seq data generated with FLI1-knockdown in two EWS cell lines (A673 and SK-N-MC at 48 h). Raw sequence reads were downloaded from SRA, mapped to reference genome (Gencode v18) using TopHat2 (40). Gene expression values were quantified with *htseq-count* command from HTSeq framework (41) and differential expression analysis was performed using DESeq2 (39) in R/Bioconductor platform. The top 200 significantly (adjusted *P*-value < 0.05, ranked by log2FC) downregulated genes upon FLI1-knockdown were analysed with i-cisTarget (using AUC threshold = 0.05) and the expected EWS-FLI1 motif was found as the first motif instance while the first ranked DHS track was on SK-N-MC cell line (Figure 2c).

In a larger study we furthermore applied i-cisTarget to 214 TF-perturbation gene signatures for 46 unique TFs (obtained from MSigDB (42)). i-cisTarget identified the correct motif for the perturbed TF for 29 of the 46 TFs and the correct TF ChIP-seq track for 25 of the datasets (for 18 TFs both the motif and ChIP-seq track are found). Note that motif and track enrichment of gene sets can also be performed through another user interface, namely the Cytoscape plugin iRegulon (8) (no web tool), which is based on the same *ranking-and-recovery* principle. iRegulon is focussed on *gene-level* analyses, whereby only motif and TF ChIP-seq enrichment analysis can be performed, allowing gene network-reconstruction. The region-based analysis in i-cisTarget on the other hand provides *CRM-level* analysis, including the detection of enriched histone marks and open chromatin tracks, which are not available in iRegulon and allows detecting specific regulatory target regions, with visualization in the UCSC Genome Browser.

## CONCLUSION

The identification of upstream regulators and their direct targets is an important question when analysing regulatory genomics data such as TF binding or open chromatin profiling data. Our tool can be used to address this question in a relatively fast and straightforward way. We demonstrated that i-cisTarget is often able to detect the correct upstream regulators when co-regulated regions are used as input, but also when co-expressed genes are used as input. An important asset of i-cisTarget is the simultaneous analysis of motif enrichment and ‘track enrichment’, being able to combine the best-scoring motifs and tracks to predict regulatory regions underlying the process under study. We believe that identifying enriched regulatory features should become as simple as identifying enriched GO terms in a gene set. As more and more data become available in public databases, it is expected that most types of input data will correlate with one or more earlier performed experiments and i-cisTarget helps to quickly find similarities between the user input data and these available datasets. Since it is based on a generic ranking-and-recovery procedure, this versatile tool can incorporate any genomic data type in the future.
